# A deep learning-assisted automatic measurement of tear meniscus height on ocular surface images and its application in myopia control

**DOI:** 10.3389/fbioe.2025.1554432

**Published:** 2025-04-11

**Authors:** Weixiao Zhang, Hua Rong, Kaiwen Hei, Guihua Liu, Meinan He, Bei Du, Ruihua Wei, Yan Zhang

**Affiliations:** Tianjin Key Laboratory of Retinal Functions and Diseases, Tianjin Branch of National Clinical Research Center for Ocular Disease, Eye Institute and School of Optometry, Tianjin Medical University Eye Hospital, Tianjin, China

**Keywords:** deep learning, tear meniscus height, automation, ocular surface image, myopia control, dry eye

## Abstract

**Purpose:**

Modalities for myopia control, such as orthokeratology, repeated low-intensity red light (RLRL) treatment, and low-concentration atropine, have become popular topics. However, the effects of these three modalities on ocular surface health remain unclear. The tear meniscus height (TMH), a crucial criterion for evaluating ocular surface health and diagnosing dry eye, is conventionally measured via manual demarcation of ocular surface images, which is inefficient and involves subjective judgment. Therefore, this study sought to establish a deep learning model for automatic TMH measurement on ocular surface images to improve the efficiency and accuracy of the initial screening of dry eye associated with myopia control modalities.

**Methods:**

To establish a model, 1,200 ocular surface images captured with an OCULUS Keratograph 5M were collected. The tear meniscus area on the image was initially marked by one experienced ophthalmologist and verified by the other. The whole image dataset was divided into a training set (70%), a validation set (20%), a test set (10%), and an external validation set (100 ocular surface images) for model construction. The deep learning model was applied to ocular surface imaging data from previous clinical trials using orthokeratology, RLRL therapy, and 0.01% atropine for myopia control. TMHs at follow-ups were automatically measured by the deep learning model.

**Results:**

Two hundred training iterations were performed to establish the model. At the 124th iteration, the IoU of the validation set peaked at 0.913, and the parameters of the model were saved for the testing process. The model IoU was 0.928 during testing. The AUC of the ROC curve was 0.935, and the R2 of the linear regression analysis was 0.92. The good performance and comprehensive validation of the model warrants its application to automatic TMH measurement in clinical trials of myopia control. There were no significant changes in the TMH during the follow-up period after treatment with orthokeratology, RLRL, or 0.01% atropine.

**Conclusion:**

A deep learning model was established for automatic measurement of the TMH on Keratograph 5M-captured ocular surface images. This model demonstrated high accuracy, great consistency with manual measurements, and applicability to the initial screening of dry eye associated with myopia control modalities.

## 1 Introduction

With the continuous increase in the prevalence of myopia worldwide, modalities for myopia control have become hotspots in research (Holden et al., 2016). Among the many modalities, orthokeratology (OK) lenses have long-term development, good safety profiles, and strong efficacy in myopic children and adolescents (Lu et al., 2024). However, its 8-h contact with the ocular surface every night raises concerns about ocular surface health, such as hypoxia, dryness, astringency, and inflammation (Xie and Wei, 2023). Moreover, great progress has been made in the repeated low-intensity red light (RLRL) therapy during the last 3 years, and this treatment uses 650-nm-wavelength red light to prevent the occurrence and control the progression of myopia. The results of clinical studies suggest that the red light therapy may act on cytochrome c oxidase in the mitochondria of choroidal vascular endothelial cells to increase choroidal thickness and boost blood perfusion in the choroid, thereby impeding myopia progression (Guo et al., 2023). However, its impact on ocular surface health remains poorly understood. Previous studies have shown that red light may serve as a therapeutic option for dry eye syndrome, improving dry eye-associated symptoms. However, the long-term effects of red light exposure on ocular surface health in children remain unclear (Antwi et al., 2024; Markoulli et al., 2021). In addition, eye drops of a muscarinic antagonist, atropine, at low concentration (0.01%), were approved by the State Food and Drug Administration in China in 2024 for delaying myopia progression in children aged 6–14 years, but the effects of topical application of atropine on ocular surface metrics, such as tear meniscus height (TMH), tear break-up time (BUT), and redness score, remain inconsistent across studies, and the outcomes are concentration-, frequency-, and duration-dependent (Cheng et al., 2020; Luo et al., 2024; Ye et al., 2022). In general, although these three modalities hold great promise for myopia prevention and control in school-aged children and adolescents, they may compromise ocular surface health, and their potential risk of eliciting dry eye needs to be evaluated objectively and accurately.

Dry eye is a multifactorial, chronic ocular surface disorder. The abnormal quality, quantity, and kinetics of tears cause tear film instability or ocular surface microenvironment imbalance, which is usually accompanied by ocular surface inflammation, tissue damage, and nerve degeneration, leading to ocular discomfort and visual dysfunction (Mohamed et al., 2022). At present, no single clinical indicator is considered the gold standard for dry eye diagnosis. Multiple tear metrics, such as tear film BUT, TMH, and tear osmolarity, as well as questionnaires (Stapleton et al., 2017; Wolffsohn et al., 2017), are necessary for a definite diagnosis. Among them, TMH measurement can effectively evaluate whether tear volume is sufficient to maintain a normal microenvironment on the ocular surface; moreover, it is relatively simple and does not involve invasive procedures, thus avoiding operation error, excessive tear secretion, and measurement inaccuracy (Markoulli and Hui, 2019). In the current clinical setting, the OCULUS Keratograph 5M, an advanced corneal topographer, is widely employed in examining ocular surface conditions and measuring the TMH (Alfaro-Juárez et al., 2019). Images of the ocular surface were captured in infrared light mode, and the TMH was manually labeled by an examiner to obtain a specific value. However, manual labeling is not only labor intensive and time consuming but also involves subjective judgment; hence, it may involve low efficiency in measurement and generate large deviations in results.

Deep learning is a type of machine learning that empowers computers to learn from experience and understand the world in a hierarchical manner (Shamshirband et al., 2021). The key technologies of deep learning involve deep neural network structures such as artificial neural networks, convolutional neural networks (CNNs), and recurrent neural networks (Choi et al., 2019; Marya et al., 2021). With improvements in hardware and algorithms, deep learning has been widely applied in the fields of ophthalmology, such as image-based diagnosis of diabetic retinopathy (Nielsen et al., 2019), retinopathy of prematurity (Gensure et al., 2020), pterygium (Xu et al., 2021), and cataracts (Zhang et al., 2019). This study sought to develop a deep learning model to automatically identify the tear meniscus area on ocular surface images and measure its height, aiming to improve the efficiency and accuracy of clinical dry eye screening associated with myopia control modalities.

## 2 Methods

### 2.1 Data collection

**FIGURE 1:**
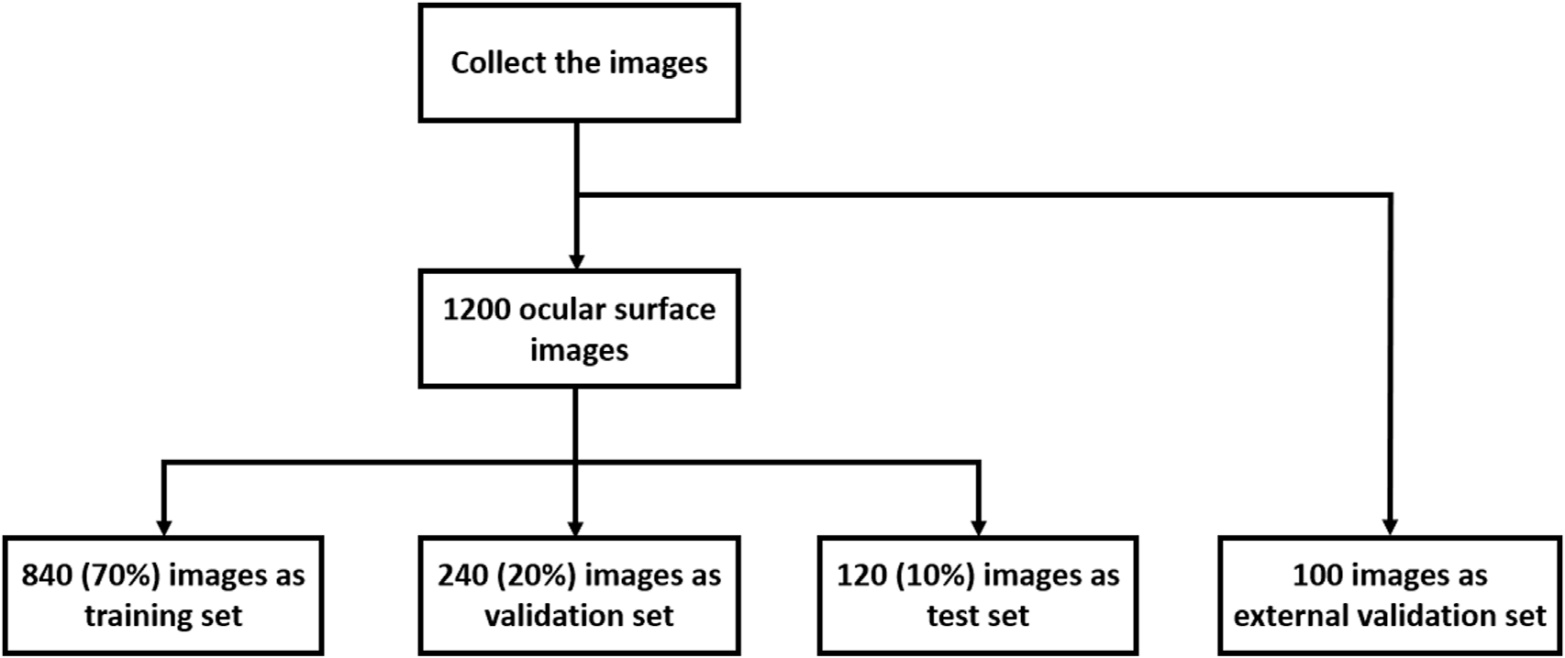
Collection and allocation of the total image dataset.

A total of 1,200 ocular surface images were randomly collected from the records of clinical examinations conducted from February 2023 to October 2023 at the Optometry Center of Tianjin Medical University Eye Hospital. All procedures in this study conformed to the principles of the Declaration of Helsinki and were approved by the Ethics Committee of Tianjin Medical University Eye Hospital. Written informed consent for participation was not required from the participants or the participants’ legal guardians/next of kin in accordance with the national legislation and institutional requirements. The images were taken with a Keratograph 5M (OCULUS, Wetzlar, Germany) and were initially screened by two experienced ophthalmologists to ensure image quality. Images where the cornea and eyelid were hard to identify as a result of ocular trauma, lower eyelid tumors, photo blur, uneven brightness, and difficulty in marking the tear meniscus area were excluded. Next, the tear meniscus area of each image was manually labeled and annotated by one of the ophthalmologists on the basis of their clinical experience, and the TMH on the ocular surface images was directly marked. These annotations were then reviewed and verified by another ophthalmologist. Agreement on the labeling and annotation must be reached between them before the image was sent for model training. These processes ensured the accuracy, reliability, and consistency of the images used in the study. The whole image set was divided into a training set (70%), a validation set (20%), and a test set (10%) (Figure 1). In addition, one hundred ocular surface images were randomly collected from the clinical examination records from March 2024 to March 2025 at the Jinnan Branch Center of Tianjin Medical University Eye Hospital as an external validation cohort.

### 2.2 Model establishment

**FIGURE 2:**
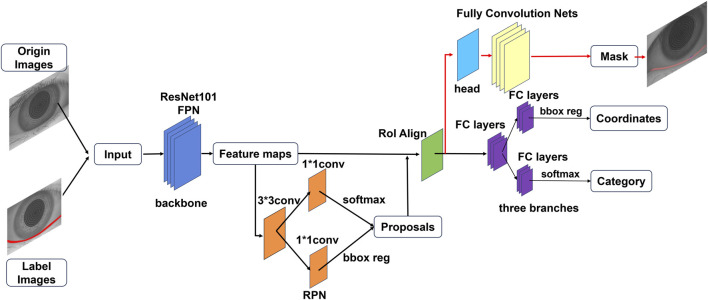
Flowchart of the deep learning model for automatic TMH measurement on ocular images.

To develop an automatic model for measuring the TMH, this study employs the Mask R-CNN model, which is composed of three primary components: a region proposal network (RPN), a feature pyramid network (FPN), and a mask segmentation branch. By utilizing the ResNet-101 backbone for feature extraction, the Mask R-CNN model further incorporates the FPN module to enhance multiscale feature representation (Figure 2).

### 2.3 Training process

During training, the annotated images were fed into the Mask R-CNN model, which was optimized via stochastic gradient descent (SGD) with a learning rate of 0.001, a momentum of 0.9, and a weight decay of 0.0005. In addition, a learning rate scheduler was applied to reduce the learning rate by a factor of 0.2 every 40 epochs, enabling improved convergence and performance of the model. The training process was conducted on a computational setup consisting of an NVIDIA GeForce RTX 3080 Ti GPU and an AMD Ryzen 5 7600X CPU.

### 2.4 Model evaluation

The intersection over union (IoU), R^2^ value, receiver operating characteristic (ROC) curve, area under the curve (AUC), and Youden index were used to evaluate model performance (Verhelst et al., 2021).

The IoU (Equation 1) describes the ratio of the intersection and union between the actual measurement results and the model prediction results. The value of the IoU ranges from 0 to 1, and the closer the value is to 1, the better the performance of the model. In this study, the IoU is used to evaluate the degree of overlap between the predicted mask and the actual tear meniscus area. The formula for IoU calculation is as follows:

$$\text{IoU}=\frac{A\cap B}{A\cup B}$$ (1)

The *R* ^2^ value (Equation 2), also known as the coefficient of determination, is an indicator of the regression performance of a model. It represents the proportion of the variance in the dependent variable Y (actual labeled value) that is explained by the independent variable X (model’s predicted value). In this study, the *R* ^2^ value is used to evaluate how closely the model’s predicted TMH aligns with the actual labeled TMH. A higher R^2^ value indicates a model prediction with greater accuracy.

$$R^{2}=\frac{\text{sum}\;\text{squared}\;\text{regression}\;\left(\text{SSR}\right)}{\text{total}\;\text{sum}\;\text{of}\;\text{squares}\;\left(\text{SST}\right)}$$ (2)

The ROC curve reflects the relationship between sensitivity (Equation 3) and specificity (Equation 4). The AUC represents the accuracy of the model, with a higher AUC indicating a larger area under the ROC curve and thus greater accuracy. In this study, the ROC curve and AUC were used to assess the model’s ability to differentiate dry eye based on the measured TMH.

$$\text{Sensitivity}=\frac{\text{TP}}{\text{TP}+\text{FN}}$$ (3)

$$\text{Specificity}=\frac{\text{TN}}{\text{TN}+\text{FP}}$$ (4)

(TP: true positive; TN: true negative; FP: false positive; FN: false negative).

The Youden index (Equation 5) is also called the correct index. The higher the value is, the greater the authenticity.

$$\text{Youden index}=\frac{\text{TP}}{\left(\text{TP}+\text{FN}\right)}+\frac{\text{TN}}{\left(\text{TN}+\text{FP}\right)}-1$$ (5)

### 2.5 Measurement of tear meniscus height

**FIGURE 3:**
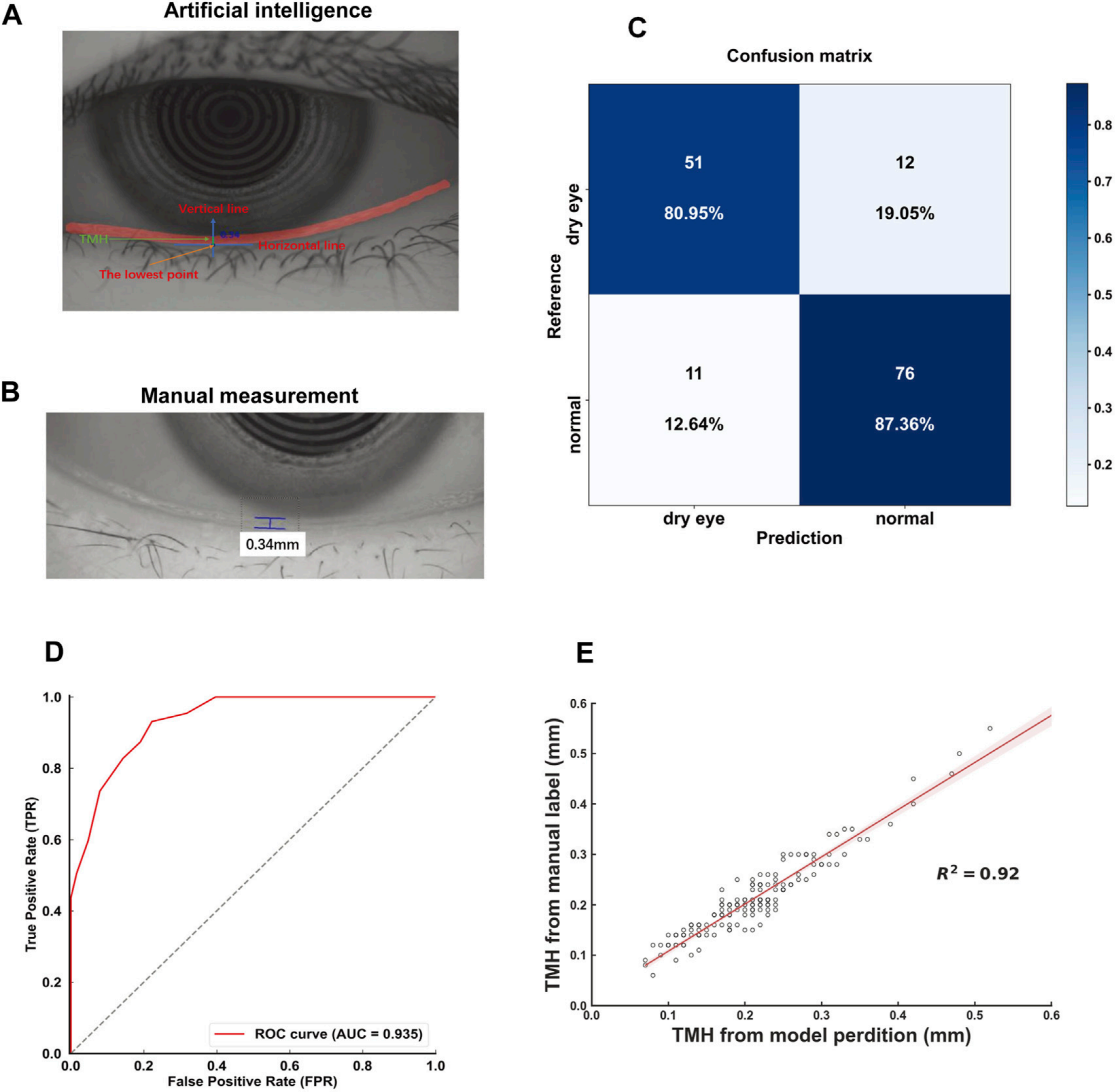
Characterization and validation of the deep learning model **(A)**. Representative picture of automatic measurement of TMH by the deep learning model. The automatic measurement of TMH (green line) involves identifying the lowest point of the tear meniscus area (red curve), drawing a vertical line (blue line) perpendicular to the horizontal line (blue line), and measuring the length of this line intercepted by the tear meniscus area (red curve) **(B)** Representative picture of the TMH manually labeled and measured by an experienced ophthalmologist **(C)** The confusion matrix for tear meniscus segmentation **(D)** An ROC curve was derived from the test set **(E)** Linear regression analysis comparing the model-assisted automatic measurement with manual measurement.

After the model was trained, a mask was generated for the identified tear meniscus area in the image. Owing to the gravity and fluidity of the tear film, the lowest point of the mask was selected, and the horizontal line passing through the lowest point was set as the x-axis. Then, a line perpendicular to the x-axis was drawn through the lowest point. The length of the vertical line segment within the mask was defined as the TMH. A schematic diagram of the tear meniscus measurement is shown in Figure 3A.

### 2.6 Clinical applications

To demonstrate the applicability of the established deep learning model in clinical practice, the imaging data of the participants who had been enrolled in previous clinical trials for the prevention and control of myopia, including the trial with orthokeratology (OK lenses), the RLRL therapy trial, and the low concentration atropine trial, at the Optometry Center of Tianjin Medical University Eye Hospital were collected for deep learning-assisted automatic measurement of the TMH. The OK lens clinical trial enrolled participants with spherical refractive errors of −1.00 to −6.00 DS, cylindrical errors of 0 to −1.5 DC, no prior OK or regular contact lens use, and best-corrected visual acuity ≥0.8. The exclusion criteria included ocular diseases, systemic conditions such as diabetes, and the use of medicines such as antihistamines, antidepressants, and diuretics. The participants underwent optical biometric measurements, corneal endothelial cell counts, and topography for customized lenses. They wore lenses nightly for 8–10 h and were followed up at 6 and 12 months after the OK lenses were worn. The RLRL therapy trial recruited participants with myopia (−0.50 to −6.00 D), astigmatism <2.50 D, and best-corrected vision ≥1.0. The exclusion criteria included ocular diseases, systemic conditions, such as albinism, psoriasis, nephrotic syndrome, systemic lupus erythematosus, and diabetes, and recent myopia control interventions. The participants underwent eye exams, wore orthotic glasses, and received red light therapy twice daily for 3 min, with follow-ups at 6 and 12 months after the beginning of the therapy. The low concentration atropine trial included children aged 6–13 years with myopia (−0.75 to −6.00 D), astigmatism <1.50 D, and best-corrected vision ≥0.8. The exclusions included strabismus, glaucoma or a tendency toward glaucoma (shallow anterior chamber, angle stenosis), allergies to scopolamine components, systemic conditions, such as albinism, traumatic brain injury, arrhythmia, congestive heart failure, coronary heart disease, and mitral stenosis, and recent myopia control interventions. Participants were randomized to receive 0.01% atropine once nightly or twice daily, with follow-ups at 3, 6, and 12 months after the initial drug administration. All procedures in these clinical trials conformed to the principles of the Declaration of Helsinki and were approved by the Ethics Committee of Tianjin Medical University Eye Hospital. The trials were registered at ClinicalTrials.gov (ChiCTR1900021759, ChiCTR2300075398, ChiCTR2200055532). Written informed consent for participation was not required from the participants or the participants’ legal guardians/next of kin in accordance with the national legislation and institutional requirements.

### 2.7 Statistical analyses

Statistical analyses were performed via SPSS statistical package 25 (SPSS, IBM, Chicago, IL, USA). For all the participants, only right-eye data were included in the analysis. Since each set of data has a sample size greater than 50, the normality of the data was assessed via the Kolmogorov‒Smirnov test. Continuous variables with a normal distribution are expressed as the mean ± standard deviation, and those with a nonnormal distribution are expressed as the median [interquartile range]. The TMH, BUT_first_, BUT_ave_, and redness scores at the follow-ups were compared with those at baseline, and the differences were examined via repeated measures ANOVA followed by Bonferroni *post hoc* tests. The changes in axial length (from baseline to the 12-month follow-up) under different myopia control modalities were compared via repeated measures ANOVA followed by Bonferroni *post hoc* tests. A P value less than 0.05 was considered statistically significant.

## 3 Results

### 3.1 Model validation

A total of 200 iterations were performed, with the IoU of the validation set peaking at 0.913 during the 124th iteration. The model parameters at this iteration were saved for subsequent testing. For the test set, the model achieved an IoU of 0.928, demonstrating its robustness. The deep learning model-assisted identification and measurement of the TMH were illustrated and compared with the conventional measurement of the TMH in a clinical setting that involves manual demarcation (Figures 3A, B). The red line in the deep learning model-assisted TMH measurement image indicates the tear meniscus area segmented by the model (Figure 3A). The confusion matrix for dry eye classification based on the TMH automatically measured from the tear meniscus region segmented by this model is shown in Figure 3C.

According to the clinical guidelines (Wang et al., 2023), TMH <0.20 mm is set as a diagnostic criterion for dry eye, and the results of deep learning-assisted automatic TMH measurement from the test set were compared with those of manual TMH measurement by experienced ophthalmologists, from which an ROC curve with an AUC of 0.935 was generated (Figure 3D). Moreover, linear regression analysis revealed a high degree of correlation between the model-assisted measurement of TMH and the manual measurement of TMH, with an *R* ^2^ value of 0.92 (Figure 3E). In addition, the Youden index was calculated on the basis of sensitivity and specificity. When the cutoff value of TMH was set at 0.20 mm, the Youden index reached 1.696, indicating the excellent performance and reliability of this deep learning model for TMH measurement.

**FIGURE 4:**
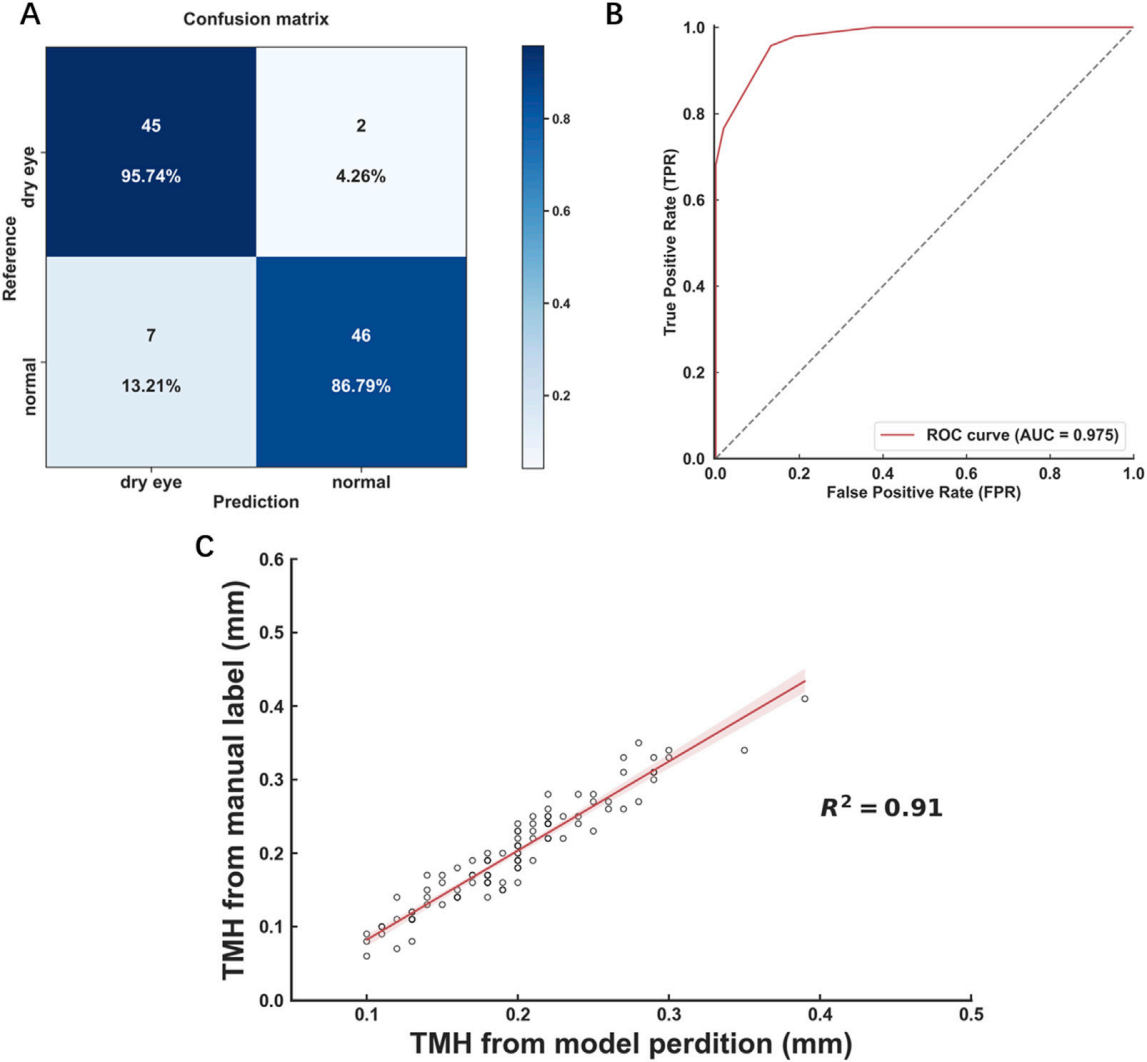
Performance of the model in external validation **(A)** Confusion matrix for tear meniscus segmentation **(B)** ROC curves derived from the external validation data set **(C)** Linear regression analysis comparing the model-assisted automatic measurement with manual measurement.

In the external validation set, the model achieved a confusion matrix for the diagnosis of dry eye (Figure 4A). The AUC of the ROC curve was 0.975 (Figure 4B). Moreover, the *R* ^2^ value of the linear regression analysis was 0.91 (Figure 4C). These results corroborated the accuracy and reliability of the deep learning model-assisted measurement of the TMH.

### 3.2 Clinical applicability

Given the satisfactory performance on the test set, deep learning model-assisted automatic measurement was applied to retrospective analyses of changes in TMH during the follow-ups of the three clinical trials using OK lenses, RLRL therapy, and low-concentration atropine for myopia control. A total of 320 myopic participants were included in the analysis of the clinical applicability of this model, with 80 participants (80 eyes) in the OK lens group, 80 participants (80 eyes) in the RLRL group, 80 participants (80 eyes) in the 0.01% atropine qd group, and 80 participants (80 eyes) in the 0.01% atropine bid group. The consistency between the model and the manual measurements of the TMH was examined in these groups (Supplementary Figure S2).

#### 3.2.1 Orthokeratology

**FIGURE 5:**
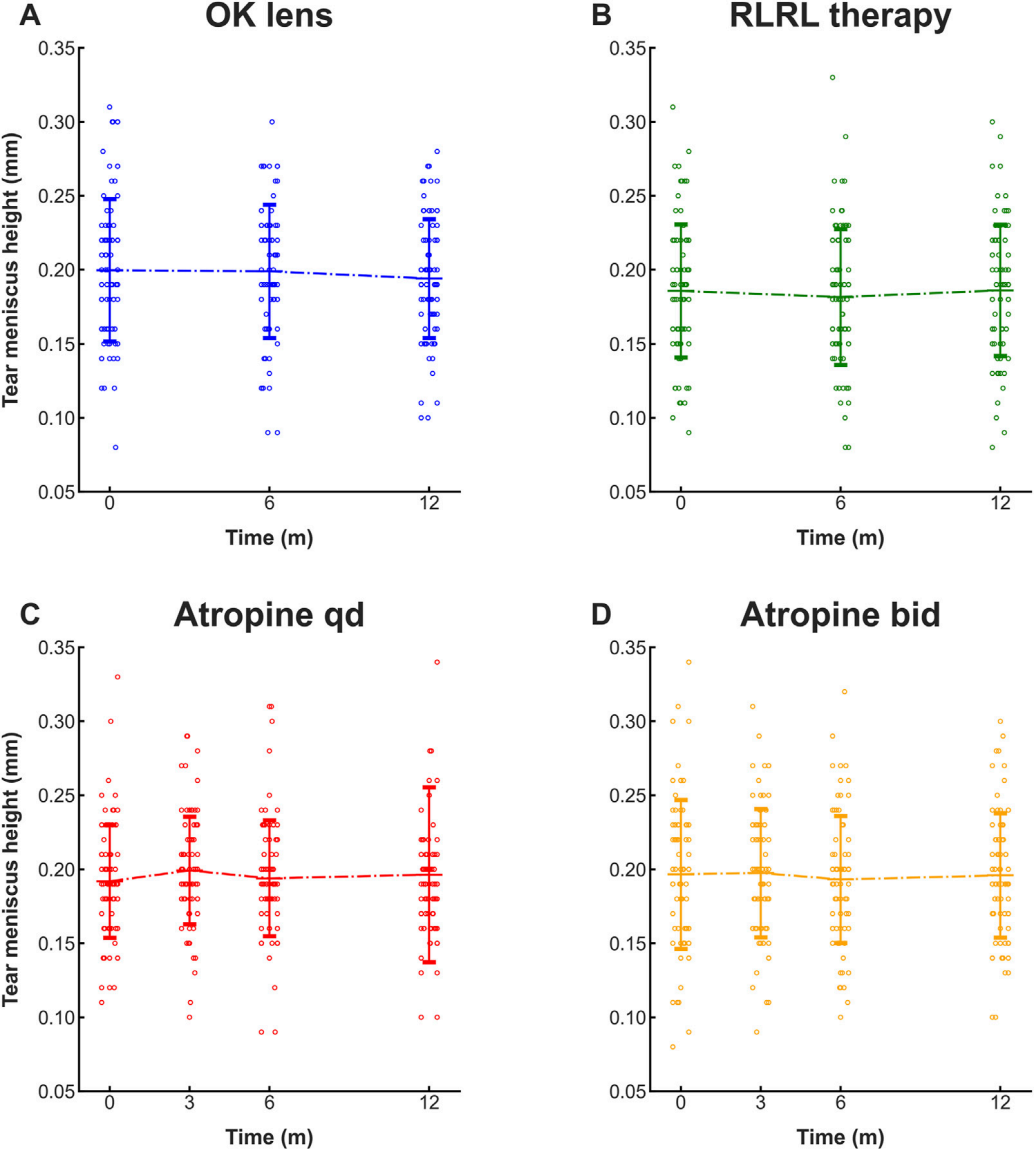
Deep learning-assisted automatic measurement of the TMH at follow-ups of clinical trials for myopia control. The TMHs automatically measured at the follow-ups of the clinical trials via the OK lens **(A)**, RLRL therapy **(B)**, 0.01% atropine qd **(C)**, and 0.01% atropine bid **(D)** were plotted and compared. N = 80. All the data are presented as the means ± SDs. OK lens: orthokeratology lens; RLRL therapy: repeated low-intensity red light; qd: once a day; bid: twice a day.

The baseline age of the participants in the OK lens group was 9.89 ± 2.56 years. The TMH was measured by the deep learning model. The baseline TMH value was 0.20 ± 0.05 mm. The TMH values at 6 months and 12 months after OK lens wearing were 0.20 ± 0.05 mm and 0.19 ± 0.04 mm, respectively (Figure 5A; Table 1). Repeated measures ANOVA revealed no significant difference in the TMH over time (all P > 0.05, for TMH at 0 m vs. TMH at 6 m, TMH at 0 m vs. TMH at 12 m, and TMH at 6 m vs. TMH at 12 m), indicating that wearing OK lenses during the 1-year period did not affect tear secretion of the participants. Similarly, no significant changes were detected in the BUT or redness score during the follow-up period (Table 1), which suggests that 1 year of OK lens wearing did not significantly affect tear film stability or the ocular surface inflammatory microenvironment (Table 1).

**TABLE 1:** Demographic information and ocular biometrics of the OK lens group.

Parameters	Baseline	6-m	12-m	P Value
Age (y)	9.89 ± 2.56	-	10.89 ± 2.56	-
IOP (mmHg)	17.34 ± 2.71	17.58 ± 2.63	17.76 ± 2.44	0.616
AL (mm)	24.93 ± 1.51	24.99 ± 1.51	25.13 ± 1.52	0.001*
TMH (mm)	0.20 ± 0.05	0.20 ± 0.05	0.19 ± 0.04	0.084
BUT_first_ (s)	9.79 ± 5.59	10.62 ± 6.53	10.41 ± 5.46	0.423
BUT_ave_ (s)	12.45 ± 5.89	13.41 ± 6.18	14.18 ± 5.92	0.641
Redness score	0.58 ± 0.29	0.57 ± 0.27	0.59 ± 0.47	0.886

IOP, intraocular pressure; AL, axial length; TMH, tear meniscus height; BUT_first_, first tear film break-up time; BUT_ave_, average tear film break-up time.

#### 3.2.2 RLRL therapy

The baseline age of the participants in the RLRL treatment group was 11.16 ± 3.14 years. The deep learning model-measured TMH at baseline was 0.19 ± 0.04 mm, and at 6 months and 12 months after RLRL therapy, it was 0.18 ± 0.05 mm and 0.19 ± 0.04 mm, respectively (Figure 5B; Table 2). No statistically significant changes were found in the TMH over time (all P > 0.05, for TMH at 0 m vs. TMH at 6 m, TMH at 0 m vs. TMH at 12 m, and TMH at 6 m vs. TMH at 12 m), indicating that the tear secretion of the participants was not affected by 1 year of RLRL therapy. Similarly, no significant changes were detected in the BUT or redness score during the same time period (Table 2), suggesting that 1 year of RLRL therapy did not significantly impact tear film stability or the ocular surface inflammatory microenvironment (Table 2).

**TABLE 2:** Demographic information and ocular biometrics of the RLRL therapy group.

Parameters	Baseline	6-m	12-m	P Value
Age (y)	11.16 ± 3.14	-	12.16 ± 3.14	-
IOP (mmHg)	17.33 ± 2.37	17.36 ± 2.61	17.59 ± 2.43	0.072
AL (mm)	24.93 ± 1.50	24.92 ± 1.49	24.98 ± 1.50	0.001*
TMH (mm)	0.19 ± 0.04	0.18 ± 0.05	0.19 ± 0.04	0.297
BUT_first_ (s)	8.37 ± 5.69	9.27 ± 5.29	9.75 ± 5.10	0.450
BUT_ave_ (s)	12.04 ± 6.18	12.48 ± 5.99	13.39 ± 5.55	0.343
Redness score	0.58 ± 0.29	0.56 ± 0.31	0.48 ± 0.26	0.281

IOP, intraocular pressure; AL, axial length; TMH, tear meniscus height; BUT_first_, first tear film break-up time; BUT_ave_, average tear film break-up time.

#### 3.2.3 Low-concentration atropine

The low-concentration (0.01%) atropine qd group had a baseline age of 10.14 ± 2.14 years. The deep learning model-measured TMH remained similar at baseline (0.19 ± 0.03 mm), 3-month follow-up (0.20 ± 0.04 mm), 6-month follow-up (0.19 ± 0.03 mm), and 12-month follow-up (0.19 ± 0.03 mm), with no statistical significance among them (Figure 5C; all P > 0.05), indicating that once-daily topical administration of 0.01% atropine for 1 year did not affect the tear secretion of the participants. Likewise, no significant changes in the BUT or redness score were detected at the follow-ups (Table 3), suggesting that the application of 0.01% atropine at this frequency did not significantly affect tear film stability or ocular surface inflammation (Table 3).

**TABLE 3:** Demographic information and ocular biometrics of the 0.01% atropine qd group.

Parameters	Baseline	3-m	6-m	12-m	P Value
Age (y)	10.14 ± 2.14	-	-	11.14 ± 2.14	-
IOP (mmHg)	17.63 ± 2.20	17.68 ± 2.33	18.28 ± 2.16	18.04 ± 2.00	0.299
AL (mm)	25.67 ± 1.67	25.79 ± 1.68	25.90 ± 1.68	26.07 ± 1.71	0.010*
TMH (mm)	0.19 ± 0.03	0.20 ± 0.04	0.19 ± 0.03	0.19 ± 0.03	0.517
BUT_first_ (s)	10.26 ± 4.98	10.12 ± 4.24	11.08 ± 4.81	11.72 ± 4.97	0.354
BUT_ave_ (s)	13.32 ± 5.33	15.04 ± 4.78	14.56 ± 5.15	15.26 ± 5.19	0.481
Redness score	0.59 ± 0.34	0.58 ± 0.22	0.58 ± 0.33	0.55 ± 0.36	0.529

IOP, intraocular pressure; AL, axial length; TMH, tear meniscus height; BUT_first_, first tear film break-up time; BUT_ave_, average tear film break-up time.

The baseline age of the participants in the low-concentration (0.01%) atropine bid group was 11.08 ± 2.59 years. The model-measured TMH and other parameters of the ocular surface followed similar trends to those of the atropine qd group, indicating that topical administration of low-concentration atropine twice daily for 1 year did not generate deleterious effects on tear secretion, tear film stability, or the ocular surface (Figure 5D; Table 4).

**TABLE 4:** Demographic information and ocular biometrics of the 0.01% atropine bid group.

Parameters	Baseline	3-m	6-m	12-m	P Value
Age (y)	11.08 ± 2.59	-	-	12.08 ± 2.59	-
IOP (mmHg)	17.54 ± 2.51	17.68 ± 2.48	17.85 ± 2.35	17.83 ± 2.10	0.302
AL (mm)	24.98 ± 1.30	25.11 ± 1.29	25.21 ± 1.29	25.34 ± 1.29	0.007*
TMH (mm)	0.20 ± 0.05	0.20 ± 0.04	0.19 ± 0.04	0.20 ± 0.04	0.310
BUT_first_ (s)	9.49 ± 5.87	9.57 ± 5.35	9.56 ± 4.77	9.77 ± 4.59	0.871
BUT_ave_ (s)	12.70 ± 5.82	13.32 ± 5.73	13.41 ± 4.93	13.56 ± 4.82	0.466
Redness score	0.57 ± 0.29	0.55 ± 0.29	0.48 ± 0.25	0.49 ± 0.25	0.419

IOP, intraocular pressure; AL, axial length; TMH, tear meniscus height; BUT_first_, first tear film break-up time; BUT_ave_, average tear film break-up time.

#### 3.2.4 Comparison of the efficacies of myopia control modalities

To compare the efficacies of different myopia control modalities, the changes in axial length (from baseline to the 12-month follow-up) of the participants in the OK lens, RLRL therapy, 0.01% atropine qd, and 0.01% atropine bid interventions were compared. The results revealed that the RLRL therapy had the most dramatic effect on controlling the increase in axial length, with the extension being merely 0.046 mm during the 1-year intervention period (Supplementary Table S1). The group of participants wearing OK lenses presented greater increases in axial length than the RLRL therapy group did (Supplementary Table S1, P < 0.001). The atropine groups presented the greatest increases in axial length, particularly the atropine qd group, which presented an increase in axial length of 0.397 mm (Supplementary Table S1).

## 4 Discussion

The current study established a deep learning model for automatic measurement of the TMH on ocular surface images to improve the efficiency and accuracy of screening for dry eye associated with myopia control modalities. In a prior study, Deng et al. (2021) developed a U-Net-based fully convolutional neural network (FCNN) to analyze ocular surface images captured by Keratograph 5M, aiming to achieve precise TMH measurements. Their study involved 242 subjects and 528 ocular surface images; however, an internal validation set and an external validation cohort were lacking for hyperparameter tuning and generalizability assessment, respectively (Yousef et al., 2023). Our study incorporates a significantly larger image dataset with a more rigorous design, including dedicated training, validation, and test sets, as well as an independent external validation cohort. This comprehensive approach not only enables more robust model training through increased data volume but also suggests greater potential for clinical applicability through external validation, which is critical for evaluating the real-world performance of deep learning algorithms in the field of ophthalmology. Moreover, two other research groups (Wang et al., 2023; Wan et al., 2023) reported deep learning-based methods for measuring the TMH in ocular surface images taken via Keratograph 5M. Both methods involve determination of the corneal center and selection of the point perpendicularly downward from the center. However, these methods may not be able to measure TMH precisely if the lower edge of the eyelid is tilted right below the corneal center, a condition that is frequently encountered in clinical settings (Supplementary Figure S2). In contrast, the deep learning model established in our study selects only the lowest point of the identified tear meniscus area. According to the principles of gravity and fluidity, the tangent line at the lowest point of the tear meniscus curve should be, or at least very close to, horizontal (Zubkov et al., 2013). This horizontal line was then set at the x-axis, a line passing through the lowest point and perpendicular to the x-axis was drawn, and the vertical line segment within the tear meniscus area was defined as the TMH (Figure 3A). Moreover, validation tests revealed an AUC of 0.935 and an *R* ^2^ of 0.92 in the ROC curve and linear regression analyses, respectively (Figures 3D, E), demonstrating the high accuracy of the model and high consistency between the model prediction value and the manual measurement value. These results also suggest that the application of this model may avoid subjectivity in TMH labeling and poor consistency among examiners. Indeed, the applications of the deep learning model to automatic TMH measurement in the imaging data of myopia control clinical trials using OK lenses, RLRL therapy, and 0.01% atropine (Figure 5) suggest the potential of this model to improve clinical work efficiency and obtain more accurate measurement results.

From the algorithmic perspective, SGD was used to optimize the model, and the learning efficiency was set to 0.001, the momentum was set to 0.9, and the weight attenuation was set to 0.0005. To enhance the convergence of the model, a learning rate scheduler was applied to reduce the learning rate by a factor of 0.2 every 40 iterations. This can improve the situation in which the algorithm easily falls into the local optimal solution.

The three modalities of myopia control involved in this study entail direct contact with the ocular surface via physical or chemical materials; however, ocular surface health should not be compromised during the myopia control. TMH is a convenient and accurate metric for diagnosing dry eye disease and evaluating ocular surface health. Among the three modalities, orthokeratology has been employed for myopia control for more than 2 decades, and the majority of the literature has not reported significant changes in TMH after long-term (>6 months) wearing of OK lenses (Li et al., 2018; Tao et al., 2021; Hou et al., 2023; Singh et al., 2020; Xie et al., 2018). The insignificant changes in the TMH measured by the deep learning model at the follow-ups postorthokeratology were consistent with the literature reports (Figure 5A; Table 1), suggesting the reliability and clinical applicability of the deep learning model. In contrast, following topical administration of atropine, changes in ocular surface metrics, such as TMH, are controversial (Cheng et al., 2020; Simonaviciute et al., 2024), and the results vary depending upon the concentration (Luo et al., 2024), frequency (Yang et al., 2024), and duration (Li et al., 2024) of drug administration. Therefore, imaging data were collected from the participants who had received topical administration of 0.01% atropine once or twice daily for a year, and the TMH at the 3-, 6-, and 12-month follow-ups was measured via the deep learning model. Moreover, RLRL therapy, an emerging modality, has attracted considerable attention in recent years. Clinical trials have focused on its efficacy (Chen et al., 2022; Xiong et al., 2022) and mechanisms (Zhu et al., 2023) for attenuating refractive power and delaying axial elongation in school-aged myopes. Indeed, the results of this study revealed the superior efficacy of RLRL therapy in controlling axial length increase compared with OK lenses and low concentration atropine (Supplementary Table S1); however, the potential side effects of RLRL therapy on ocular surface health remain unknown. To our knowledge, this study, for the first time, reported that no significant changes were detected in the TMH, BUT_first_, and BUT_ave_ of the myopic participants that had received RLRL therapy, indicating that RLRL therapy did not affect tear secretion and tear film stability and thus might be a promising myopia control modality with both efficacy and safety. More importantly, the insignificant changes in the TMH at the follow-ups of RLRL therapy (Figure 5B; Table 2) and 0.01% atropine topical administration (Figures 5C, D; Tables 3, 4) support the application of the deep learning model for automatic measurement of the TMH and implicate the ocular surface safety profiles of these myopia control modalities.

This study has several limitations. First, the number of samples was limited, and 1,200 ocular surface images captured via Keratograph 5 M may lead to sample selection bias due to the unequal distribution of dry eye cases. Moreover, although a validation set comprising 10% of the images was used and good results were obtained, more diverse ocular surface images are needed to increase the versatility of the deep learning models.

In summary, the deep learning model established in this study can automatically identify the tear meniscus area and measure the TMH on the basis of ocular surface images taken via Keratograph 5M. This model demonstrates high accuracy and great consistency with manual measurements, as well as applicability to the initial screening of dry eye associated with myopia control modalities.

## Data Availability

The raw data supporting the conclusions of this article will be made available by the authors, without undue reservation.
