# Supplementary material for: A deep learning-assisted automatic measurement of tear meniscus height on ocular surface images and its application in myopia control
Source: Front Bioeng Biotechnol. 2025 Apr 11;13:1554432. doi: 10.3389/fbioe.2025.1554432 (PMC12021850; doi:10.3389/fbioe.2025.1554432)
Supplement: Supplementary file 1 [file DataSheet1.pdf]

Supplementary material

Supplementary figures

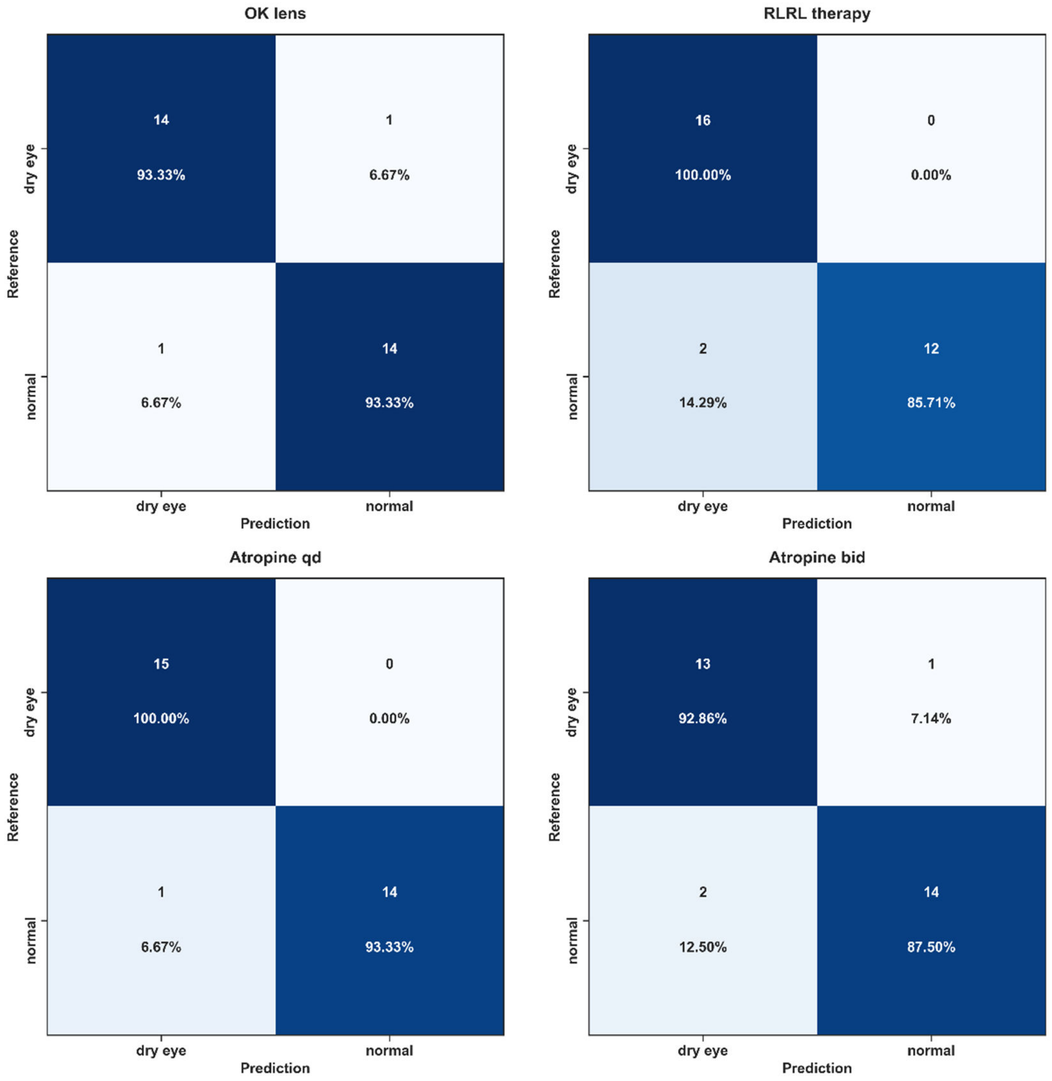

Supplementary figure 1. Confusion matrices of the model for examining the consistency of dry eye diagnosis in the OK lens, RLRL therapy, 0.01% atropine qd, and 0.01% atropine bid groups, each consisting of 30 participants.

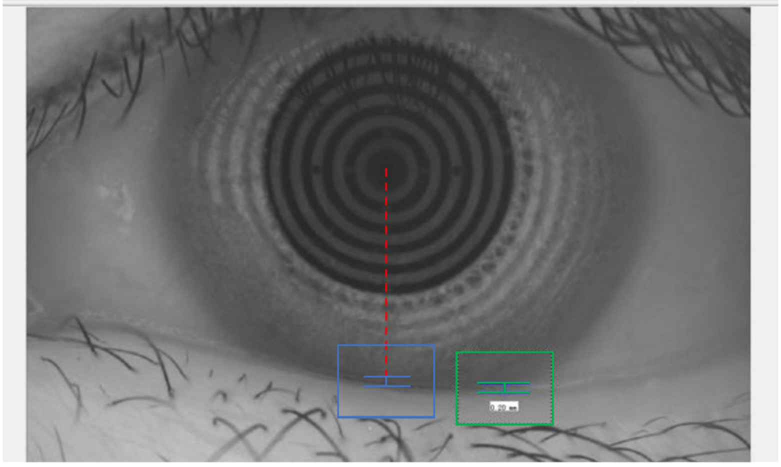

Supplementary figure 2. Comparison between the TMH measured by the model established in this study and that measured by the models established in other studies. The green box indicates the TMH measured by the deep learning model established in this study. The models proposed by Wan et al. and Wang et al. determine the TMH by drawing a vertical line (red dotted line) downward from the corneal center; however, the measurement frame (blue lines) does not align with the lower eyelid edge, which may cause measurement inaccuracy.

Supplementary table

Supplementary Table 1. Comparison of axial length changes (baseline to the 12-month follow-up) under different modalities of myopia control

Modality 1 vs Modality 2	Axial length changes from baseline to the 12-month follow-up (mean ± SD, mm)		P value
	Modality 1	Modality 2	
RLRL vs. OK	0.046±0.097	0.202±0.080	＜0.001*
RLRL vs. 0.01% atropine qd	0.046±0.097	0.397±0.152	＜0.001*
RLRL vs. 0.01% atropine bid	0.046±0.097	0.363±0.156	＜0.001*
OK vs. 0.01% atropine qd	0.202±0.080	0.397±0.152	＜0.001*
OK vs. 0.01% atropine bid	0.202±0.080	0.363±0.156	＜0.001*
0.01% atropine qd vs. 0.01% atropine bid	0.397±0.152	0.363±0.156	0.984

* $P < 0.05$, repeated measures ANOVA followed by Bonferroni correction.
